# Mast cells as novel mediators of reproductive processes

**DOI:** 10.3389/fimmu.2013.00029

**Published:** 2013-02-14

**Authors:** Katja Woidacki, Federico Jensen, Ana C. Zenclussen

**Affiliations:** Department of Experimental Obstetrics and Gynecology, Medical Faculty, Otto-von-Guericke UniversityMagdeburg, Germany

**Keywords:** mast cells, placenta, pregnancy, uterus, implantation

## Abstract

The relationship between mast cells (MCs) and pregnancy is a controversially discussed topic. The presence and quantitative distribution of MCs in the reproductive tract was confirmed in different species. A phase-dependent oscillation of MCs during the hormonal regulated estrous cycle was suggested and on this basis, MCs were assumed to play a positive role in implantation because of their ability to secrete histamine. At later pregnancy stages, they were proposed to have rather a negative role, as their exacerbated activation is associated with pre-term delivery. The present review is intended to provide an overview about uterine MCs that bring to light their unexpected relevance for reproductive processes.

## INTRODUCTION

Already in the early 60s and 70s the presence and quantitative distribution of mast cells (MCs) in the reproductive tract in general and during the estrous cycle in particular was described in different species like rat (Gibbons and Chang, 1972), hamster (Harvey, 1964), and cow (Likar and Likar, 1964). The phase-dependent oscillation of MC numbers during the hormone-regulated estrous cycle was described and assumed that they are important for implantation because they secrete substances that promote tissue remodeling necessary for this process. The functional importance of these data was however unclear as the results were based on the methodological simplicity at that time. Later on, a role for MCs in reproduction was dismissed because of the apparently normal pregnancy outcome of mice lacking MCs. We have recently investigated the importance of MC deficiency in pregnancy outcome using a mouse model. The present review is intended to provide an overview about the available as well as novel data from us and many others, that bring to light the unexpected relevance of MCs for reproductive processes.

## UTERINE MCs REPRESENT A DIVERGENT PHENOTYPE FROM OTHER DESCRIBED MCs

Based on their tissue specificity, murine MCs are typically classified as either mucosal or connective tissue-type MCs (Metcalfe et al., 1997). Thereby, this classification is attributed, i.a. to their histochemical staining patterns especially to those of the granule-stored proteases. Already in 1960, Spicer suggested that uterine MCs (uMCs) represent a divergent phenotype composed of mucosal as well as connective tissue-type MCs (Spicer, 1960). The histochemical observations were done by using a combined Alcian blue–Safranin stain whereby mucosal MCs are Alcian blue-positive and Safranin-negative and connective tissue-type MCs are Alcian blue-negative and Safranin-positive, respectively. In our laboratory we could confirm Spicer’s findings on the presence of both MC types. uMCs were positive either for Alcian blue or for safranin. In addition, we found a third MC population that was positive for both dyes (Woidacki et al., 2013). These cells were already described and reportedly reflect different stages of differentiation (Reynolds et al., 1988; Kitamura, 1989). Moreover, they can derive from or represent an ongoing transdifferentiation process, during which mature MCs may change their content in proteoglycans, amines, peptides, etc. (Michaloudi and Papadopoulos, 1999). These conversion processes might be induced as a response to local inflammatory processes (Kitamura, 1989; Tsuji et al., 1990; van Overveld, 1990; Moon et al., 2010) or the presence of fibroblasts (Levi-Schaffer et al., 1986). Here, the most important growth factor for MCs, stem cell factor (SCF), could serve as a pivotal stimulus. It was described that MC proliferation and differentiation in the uterus is regulated by SCF secretion from uterine smooth muscle cells (Mori et al., 1997b). Furthermore, it is known that heparin is essential by controlling the levels of specific granule proteases inside MCs (Humphries et al., 1999). Thereby, the tissue-specific MC phenotype can vary in different mouse strains. In BALB/c, ear MCs express MC protease 7 (Mcpt-7), the analog of human tryptase α/β 1. In contrast, ear MCs from C57BL/6J contain no detectable protein levels of Mcpt-7 (Ghildyal et al., 1994). During pregnancy, uMCs seem to maintain their heterogeneity as in the pregnant human uterus tryptase-positive and chymase-negative (MCT) as well as tryptase-positive and chymase-positive (MCTC) MCs are present (Garfield et al., 2006). In pregnant rats, uMCs have been identified likewise as mucosal and connective tissue-type MCs based on their specific protease content (Salamonsen et al., 1996). It is therefore clear that uMCs represent a heterogeneous population of cells and they can change their phenotype according to local stimuli. Thus, uMCs constitute a special population with unique characteristics and a high plasticity, which is important to consider when designing experiments to analyze their role *in utero*.

## uMCs OSCILLATE DURING THE ESTROUS CYCLE

The presence of uMCs and their menstrual or estrous cycle-related variations in number and structure were described in humans (Drudy et al., 1991a,b; Mori et al., 1997a) and other mammals like mouse (Padilla et al., 1990), rat (Aydin et al., 1998), hamster (Harvey, 1964), and goat (Karaca et al., 2008). In mouse, uMCs seem to reach their highest level during the receptive phase of the female, namely in estrus (Woidacki et al., 2013), when the uterus is prepared for nidation. This is in line with results in rats reported by Aydin et al. (1998). They detected the highest number of MCs in estrus as well. If fecundation did not occur the MC number in metestrus was decreased. After pregnancy establishment, uMC numbers became even higher (Woidacki et al., 2013). This might be due to the interplay of the sexual hormones 17β-estradiol and progesterone. In mice, maximum 17β-estradiol levels were observed at estrus whereas progesterone levels were lowest at this phase (Fata et al., 2001). Estradiol is known to potentiate the degranulation of MCs *in* *vitro *(Cocchiara et al., 1992). All these observations indicate a hormone-dependent regulation of uMCs. This is further reinforced by our recent observations. We found that not only MCs express the receptors for estrogens and progesterone but these hormones in combination can attract MCs *in vitro *and *in vivo *to uterine cells (Jensen et al., 2010).

## MAST CELLS ARE IMPORTANT FOR INDUCTION AND MAINTENANCE OF PREGNANCY

The fundament of a successful pregnancy outcome in mammals is the maternal tolerance of the semi-allogenic fetus based on a well-orchestrated modulation of the maternal immune system and the functionality of the hormonal system. A variety of innate and adaptive immune cells are participating in this concert especially locally at the feto-maternal interface including uterine natural killer cells (Greenwood et al., 2000; Bilinski et al., 2008), dendritic cells (Blois et al., 2004; Plaks et al., 2008), and regulatory T cells (Aluvihare et al., 2004; Zenclussen et al., 2006; Schumacher, 2013), whereas the function of MCs in maternal tolerance is uncertain. High amounts of MCs were detected in the uterus during pregnancy (Menzies et al., 2012) and MC density was significantly higher in tissue from pregnant women than those of non-pregnant women (Garfield et al., 2006). We confirmed high numbers of uMCs in early pregnancy stages in a mouse model. uMCs were mainly distributed between implantation sites. Implantations from MC-deficient C57BL/6J-*Kit*^W-sh/W-sh^ (W-sh), whose MC deficiency is caused by a defective *c-Kit* signaling, showed a delayed kinetic of development with a significantly diminished size in comparison to wild-type, MC sufficient controls (Woidacki et al., 2013). The transfer of bone marrow-derived MCs (BMMCs) into W-sh mice positively influenced the size of the implantation sites and restored them to normal levels (Woidacki et al., 2013). It is important to remark that a delayed implantation might have a fatal impact in pregnancy outcome (Song et al., 2002). This is further evidenced by our findings on insufficient placentation and remodeling of spiral arteries in W-sh mice (Woidacki et al., 2013). The embryo itself could act as the stimulus for the implantation process. Here, the embryo-derived histamine-releasing factor (EHRF) might be one of the first signals from the embryo to the uterus. The EHRF-induced local secretion of histamine by uMCs could play a role in preventing maternal immune rejection at the implantation stage (Cocchiara et al., 1986).

Some studies are based on histamine as an important MC-specific mediator for the initiation of blastocyst implantation processes and decidual cell responses (Shelesnyak et al.,1957, 1959; Nalbandov et al., 1971). However, the increment of the uterine histamine levels in MC-deficient WBB6F_1_-W/W^V^ (W/W^V^) after steroid treatment (Wordinger et al., 1985) suggests an alternative source of histamine like endothelial cells (Robinson-White et al., 1982) or/and decidual cells which have been shown to release histamine upon stimulation (Schrey et al., 1995). A study from Wordinger et al. (1986) makes the discussion even more controversial. The sterility of MC-deficient WBB6F-W/W^V^ is mainly due to atrophic ovaries with a hyperplastic stroma and absence of follicles and distinct corpora lutea (Wordinger et al., 1985). To determine whether implantation and live births occurred in the absence of uMCs, Wordinger et al. (1985) employed a model of ovariectomized female W/W^V ^mice. After the transplantation of one ovary obtained from normal female littermates (+/+) the authors transferred blastocysts from +/+ into pseudopregnant W/W^V^. They could not find any differences between W/W^V^ and +/+ in the implantation rate after blastocyst transfer or in the number of live births. Because of these observations, Wordinger et al. (1985) excluded any requirement of uMCs in these processes. However, the transplanted ovaries were obtained from +/+ and should therefore contain a remarkable amount of already mature and differentiated MCs that could then migrate to the surrounding tissue and expand. We recently observed that locally transferred MCs into one single uterine horn were located in the other, untreated, uterine horn shortly thereafter (Woidacki et al., 2013). In the Wordinger study, no information is available regarding the presence of MCs in the ovary before and after transplantation. Additionally, the recipients were treated with steroids and MCs are known for their susceptibility to the action of hormones like estradiol and progesterone (Wordinger et al., 1985; Cocchiara et al., 1992; Rudolph et al., 2004; Jensen et al., 2010) which probably induced the expansion of the MCs present in the ovaries and their migration to the uterus. We detected high amounts of MCs within the ovaries (unpublished observations) that coincides with observations done in different species like mouse (Skalko et al., 1968), rat (Jones et al., 1980; Gaytan et al., 1991; Aydin et al., 1998; Batth and Parshad, 2000), hamster (Shinohara et al., 1987; Krishna and Terranova, 1991), cow (Reibiger and Spanel-Borowski, 2000), goat (Karaca and Simşek, 2007; Karaca et al., 2008), and chicken (Parshad and Kathpalia, 1993). Furthermore, we could not find alterations in the number of follicles as well as corpora lutea between MC-deficient W-sh and control mice (Woidacki et al., 2013). As early pregnancy is highly dependent on the presence of corpora lutea and the progesterone they secrete, we conclude that the establishment of pregnancy does not seem to depend on MCs but implantation and embryo development surely does (Woidacki et al., 2013).

On day 10 of murine normal pregnancies, MCs were present in the decidua, the maternal part of the feto-maternal interface and located closed to blood vessels (Woidacki et al., 2013). In pregnant rats, the degranulation of MCs positively influenced angiogenesis (Varayoud et al., 2004; Bosquiazzo et al., 2007). Nevertheless, some studies exclude MCs as important mediators of pregnancy-relevant processes. Salamonsen et al. (1996) applied to syngeneically mated female rats a highly potent MC stabilizer (FPL 55618). They claimed no differences in the number of implantation sites. This is remarkable, but taking a closer look at the data, as e.g., two of a total of five rats receiving FPL intraperitoneally failed to show any embryo implantation (Salamonsen et al., 1996), meaning only 60% of the animals, got pregnant compared to more than 80% in the control group. Furthermore, the low number of animals employed it is questionable to make such strong conclusions based only in a subgroup of the studied group. Menzies et al. (2012) recently suggested that the absence of MCs had no discernible impact on pregnancy. In this study, MC-deficient C57BL/6J-*Kit*^W-sh/W-sh^ and their wild-type counterparts, both syngeneically mated, had similar offspring birth weights and no difference in fetal–placental index. However, neither the kinetic nor the occurrence of implantations was analyzed or reported by these authors. That survivor animals develop normally does not discard that the first stages of pregnancy are dependent on MCs. Despite that, this study concentrated on those pregnancies that succeed after implantation and no data is provided as to how many females were pregnant after plug detection and how many blastocysts could be implanted, these authors concentrated on syngeneic matings. These two publications denying a role of MCs in pregnancy share one aspect: they are based on syngeneic and therefore biologically questionable matings. Naturally occurring pregnancies *in natura *are predominantly allogeneic to maintain the genetical variability of a species. That allows the adaptation of the fetus to its later environment at the best. Matings with genetically related and even worse among identic individuals has to be avoided because of the partially tremendous consequences of inbreeding. Syngeneic matings are exclusively necessary to maintain inbreeding colonies in the laboratories. Even there, after some generations mostly a backcross to wild-types has to be done as a result of the genetic impoverishment. Madeja et al. (2011) could show that murine allogeneic fetuses and placentas were heavier at term compared with syngeneic controls. This consequence was based on impaired decidual vascularization as well as placental and fetal growth after syngeneic matings. They supposed that allogeneic placentas are much more sufficient in supporting fetal growth by adequate modulation of spiral arteries. It seems reasonable to assume that paternal allo-antigens are important for stimulating maternal immune cells, which is not further discussed as it would go beyond the scope of this article. In this context, the role of MCs is worth to be studied and critically analyzed in efficient, relevant allogeneic pairings as the results obtained in syngeneic ones are limited by the already mentioned factors.

MC-deficient C57BL/6J-*Kit*^W-sh/W-sh^ (W-sh) mice implanted significantly less blastocysts than their wild-type counterparts after allogeneic mating. Uteri from W-sh mice were either very thick, swollen, and reddish with no visible implantations or contained few implantations. Accordingly, their litter size was significantly reduced as compared to wild-type controls. The systemic and local reconstitution with BMMC completely restored the reproductive phenotype of W-sh mice. Moreover, the few implanted blastocysts in W-sh mice developed significantly smaller placentas and insufficient modifications of the spiral arteries that are responsible for supplying oxygen and nutrients to the fetus. BMMC transfer normalized all parameters and therefore contributed to a normal pregnancy outcome by mediating placental development and spiral artery remodeling (Woidacki et al., 2013).

**FIGURE 1:**
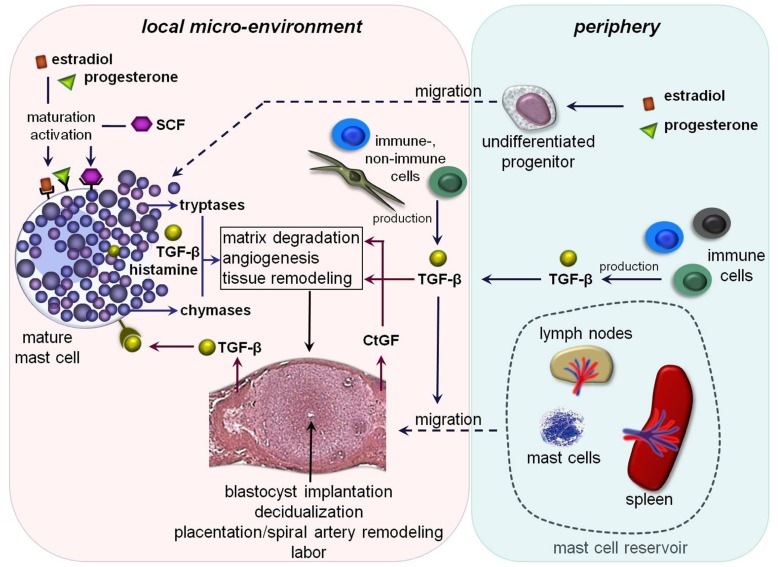
**Hypothetical scenario of MC-impact on pregnancy-related processes: undifferentiated MC-precursors migrate from the periphery into the uterus due to the simultaneous influence of estradiol and progesterone.** Their local maturation and differentiation occurs through SCF. Amongst other mediators estradiol and progesterone could bind locally on uMCs that further lead to their activation. The MC-activation results in a simultaneous release of pre-formed and/or *de novo *synthesized mediators including different tryptases and chymases as well as transforming growth factor-β (TGF-β) and histamine. These mediators could directly or indirectly affect important processes like blastocyst’s implantation, decidualization, placentation, spiral artery remodeling, and later on labor. Trophoblast-derived and/or peripheral TGF-β binds on TGF-β-receptors expressed on the mast cell surface that would be a possible further mechanism for their activation. TGF-β likewise stimulates the recruitment of other MCs from the periphery into the fetal–maternal interface. Here, lymph nodes and spleen could serve as a MC-reservoir in the periphery. Fibroblast- and later on trophoblast-derived connective tissue growth factor (CtGF) is involved in matrix degradation, angiogenesis as well as tissue remodeling.

Inadequate placental development mainly due to discrepancies in trophoblast differentiation and invasion, respectively can lead to intrauterine growth retardation (IUGR) and pre-eclampsia amongst other complications. In placentas obtained from IUGR-pregnancies the number of MCs was markedly decreased while hypoxia could intensify MC degranulation (Szukiewicz et al., 1999b). The degranulation of MCs resulted in a greater increase of the vascular resistance in pre-eclampsia likely due to the vasoconstrictive function of histamine (Szukiewicz et al., 1999a) and asthmatic pregnant women are at increased risk to develop this disease (Siddiqui et al., 2008; Murphy et al., 2011). In severe pre-eclampsia, the number of human MC chymase-positive cells was significantly higher compared to normal pregnant women (Mitani et al., 2002) and MC-chymase is known to be more potent than angiotensin-converting enzyme to convert angiotensin I to angiotensin II (Wintroub et al., 1984). Whether the secreted histamine, that inhibits the apoptotic activity in trophoblast cells via H(1) receptor (Pyzlak et al., 2010) and further influences the process of trophoblast differentiation (Szewczyk et al., 2005) and invasion (Liu et al., 2004) is derived exclusively from MCs has to be still determined. Thus, the heterogenicity of uMCs is also depicted here: low numbers of uMCs is associated with pathologies as IUGR which would predict a positive role of uMCs on fetal growth while their exacerbated activation is related to pre-eclampsia and pre-term birth. Hence, uMCs represent a heterogenous population, which shows also a high plasticity to respond differently to different stimuli.

## MCs AND THEIR INFLUENCE ON PERINATAL PROCESSES

There are strong hints for the relevance of MCs in mediating the implantation of the blastocyst as discussed above. As pregnancy advances, MCs exert an influence on the maintenance of pregnancy by allowing the unrestricted development of the placenta and remodeling of the spiral arteries (Woidacki et al., 2013). Interestingly, there are vast evidences that MCs also influence perinatal processes. The degranulation of MCs can lead to substantial changes in the myometrial contractility (Martínez et al., 1999; Garfield et al., 2006). Resident MCs increased uterine contractility in pregnant guinea pigs through multiple mediators including histamine and serotonin. Uterine responses to these mediators are dependent on gestational age (Bytautiene et al., 2008). Pregnant women affected by systemic mastocytosis exhibit manifestations of pre-term labor and delivery. This disease is accompanied by an unexplained and pathologic increase in MCs in specific tissue (Metcalfe and Akin, 2001). The allergic activation of MCs results in a substantial increase in uterine contractility (Garfield et al., 2006) and could be therefore responsible for the allergy-associated induction of pre-term labor (Habek et al., 2000; Bytautiene et al., 2004). This is in line with the fact that pregnant women with asthma are at a higher risk to pre-term delivery (Perlow et al., 1992; Sorensen et al., 2003; Murphy et al., 2006). However, Menzies et al. (2012) concluded that MCs have no impact on initiation of labor because the time of labor initiation in MC-deficient mice was indistinguishable from wild-type controls. Nevertheless, the number of MCs within the mouse cervix doubled from non-pregnant to day 18 of pregnancy, with a further 1.5-fold increase with labor (Menzies et al., 2012). This relevant question is worth to be tested in a mouse model for pre-term delivery and remains highly up-to-date.

In summary our data as well as data from the literature show that MCs accompany and deeply affect many steps of reproduction. MCs are modified and attracted by hormones, uMCs are essential for allowing implantation of allogeneic embryos, and positively influence placentation and thus, embryo development. Later on, an exacerbated number or function of uMCs can negatively influence pregnancy and foster pre-term delivery. It is clear that uMCs are not only different from other MCs because of their unique markers but also seem to secrete different mediators at different pregnancy stages and upon different stimuli. This makes these cells an extremely interesting target of study for both, reproductive biologists and MC researchers. Based on the data discussed in this review, we propose following hypothetical scenario for the impact of MCs on pregnancy-related processes.

## CONCLUSION

Mast cells vitally influence reproductive processes and in particular the pregnancy itself by modulating non-immunological responses like tissue remodeling, angiogenesis, optimal placentation, and spiral artery modifications as well as labor. They further play a rather negative role in parturition as the excessive secretion of MC-mediators may lead to pre-term delivery. MCs may act not only as mediators of the innate immune system but also as cellular switch points between innate and adaptive immune responses. Their activity is regulated by endocrine and physiological signals and based on their granule-stored array of biologically active products. All these well-orchestrated mechanisms allow the non-restrictive development of the semi-allogeneic fetus within the maternal uterus and therefore fetal survival. The understanding of the paradoxon “pregnancy” is of fundamental importance for helping couples to realize their often unfulfilled desire to have children. In this context, the data in regard to the mast cell-associated positive pregnancy outcome might serve as a further puzzle piece to answer these questions.

## Conflict of Interest Statement

The authors declare that the research was conducted in the absence of any commercial or financial relationships that could be construed as a potential conflict of interest.
